# Poisoned by Mercury from a Filling

**Published:** 1872-04

**Authors:** J. S. Rice


### POISONED BY MERCURY FROM A TOOTH FILLING.

BY J. S. RICE, M. D.

The circumstances reported under the above caption in the January number of the REGISTER, were of extraordinary interest as the embodiment of more professional blundering than one often sees in so small a space.

The subject of that Coroner's jury evidently died of phlegmonous erysipelas. The first symptoms noticed were toothache, with swelled face and neck, for the relief of which a physician (?) was called who fanned the flame by applying poultices, the inflammation of the peridental membrane extended to the maxillary periosteum, thence to the gums and other soft tissues of the buccal cavity, passed on to the fauces, and perhaps, to the glottis, and produced death by apnœa.

The direct administration of mercury in any form would not produce symptoms similar to those in so short a time, and to suppose that sufficient of the mercury contained in the filling, could evaporate, oxidize, or to be converted into a soluble salt by any influences within the mouth to produce fatal ptyalism is simply ridiculous, and is unsupported by a precedent in toxicology.

Unfortunately, Dr. Keef may not have a thorough knowledge of the dental pathology, and his breath may have had a slight odor of intoxicating beverage at the time, but he deserves no censure in connection with the death of Mr. Smith. The tooth was, no doubt, plastered up with a huge daub of amalgam, as thousands have been before; the pulp may have been alive, and largely exposed, or dead, it makes little difference which, but it is obvious that there was periodontitis when the patient complained of toothache, and swelling, and soreness of the face, and it is equally patent that the same thing would have resulted had the tooth been filled with gold or any other material that would hermetically seal the cavity, and prevent the escape of the gas arising from the decomposing pulp or its debris.

It is evident to my mind that the prompt extraction of Smith's tooth would have obviated all danger in his case.

The true verdict of that Coroner's jury should have been that "the deceased came to his death by phlegmonous erysipelas brought about by the treatment of an inflamed tooth by Drs. Sprague, Davis and Buffon."

I do not write this article as a vindication of Amalgam, it has many faults, and few advantages, and every conscientious dentist should disdain to use it, except in cases that are few and far between.

